# Big five personality traits of medical students and workplace performance in the final clerkship year using an EPA framework

**DOI:** 10.1186/s12909-024-05434-x

**Published:** 2024-04-25

**Authors:** Harm Peters, Amelie Garbe, Simon M. Breil, Sebastian Oberst, Susanne Selch, Ylva Holzhausen

**Affiliations:** 1 Dieter Scheffner Center for Medical Education and Educational Research, Deans´Office of Study Affairs, Charité - Universitätsmedizin Berlin, Campus Charité Mitte, Charitéplatz 1, 10117 Berlin, Germany; 2 Institute for Psychology, University of Münster, Münster, Germany; 3 Department of General Practice and Primary Care, University Medical Center Hamburg- Eppendorf, Hamburg, Germany

**Keywords:** Workplace performance, Entrustable professional activities, Personality traits, Big five

## Abstract

**Background:**

The qualities of trainees play a key role in entrustment decisions by clinical supervisors for the assignments of professional tasks and levels of supervision. A recent body of qualitative research has shown that in addition to knowledge and skills, a number of personality traits are relevant in the workplace; however, the relevance of these traits has not been investigated empirically. The aim of this study was to analyse the workplace performance of final-year medical students using an Entrustable Professional Activity (EPA) framework in relation to their personality traits.

**Methods:**

Medical students at the end of their final clerkship year were invited to participate in an online survey-based, cross-sectional field study. In the survey, the workplace performance was captured using a framework consisting of levels of experienced supervision and a defined set of 12 end-of-undergraduate medical training EPAs. The Big Five personality traits (extraversion, agreeableness, conscientiousness, neuroticism, and openness) of the participating medical students were measured using the Big Five Inventory-SOEP (BFI-S), which consists of 15 items that are rated on a seven-point Likert scale. The data were analysed using descriptive and inferential statistics.

**Results:**

The study included 880 final-year medical students (mean age: 27.2 years, SD = 3.0; 65% female). The levels of supervision under which the final-year clerkship students carried out the EPAs varied considerably. Significant correlations were found between the levels of experienced supervision and all Big Five dimensions The correlations with the dimensions of extraversion, agreeableness, conscientiousness and openness were positive, and that for the neuroticism dimension was negative (range *r* = 0.17 to *r* = − 0.23). Multiple regression analyses showed that the combination of the Big Five personality traits accounted for 0.8–7.5% of the variance in supervision levels on individual EPAs.

**Conclusions:**

Using the BFI-S, we found that the levels of supervision on a set of end-of-undergraduate medical training EPAs were related to the personality traits of final-year medical students. The results of this study confirm the existing body of research on the role of conscientiousness and extraversion in entrustment decision-making and, in particular, add the personality trait of neuroticism as a new and relevant trainee quality to be considered.

**Supplementary Information:**

The online version contains supplementary material available at 10.1186/s12909-024-05434-x.

## Background

The assignment of professional tasks at a certain level of autonomy to individual trainees by clinical supervisors is a central phenomenon and characteristic of workplace learning in medical education and beyond in health profession education. The concept of Entrustable Professional Activities (EPAs) draws on this tradition to conceptualize learning and assessments in the clinical workplace [1]. A growing body of research provides a better understanding of the often numerous explicit and implicit factors that influence why one trainee is entrusted with performing a specific task while another trainee is not. These factors include the personal qualities of trainees. For example, qualitative research has shown that certain personality traits of trainees play a role in supervisors’ entrustment decisions [2]. Yet, this relationship has not been systematically investigated. Such an investigation could confirm, complement and extend our understanding of which trainee qualities influence the entrustment decision-making processes of supervisors. The purpose of this study was to analyse the workplace performance of final-year medical students using an EPA framework in relation to their personality traits measured by an established Big Five questionnaire.

A valuable and practical reconceptualization of workplace performance has been achieved through an EPA framework [1]. This framework builds on three main components: (1) professional activities, i.e., authentic task characteristics of a profession; (2) levels of supervision, which indicate the proficiency of a trainee in carrying out a professional activity; and (3) entrustment decisions, which link the first two components and are made by supervisors when managing patient care [2, 3]. Within the EPA framework, workplace performance increases by the number of EPAs a trainee can carry out and the level of autonomy, a trainee can carry out a professional tasks. Consider, for example, two trainees in their final year of study who have the same supervisor. Trainee A is regularly entrusted with taking medical histories independently, with only the key findings checked afterwards. For Trainee B, the supervisor takes on a much more active role, and the medical histories are checked together with the supervisor. Why are some trainees trusted more than others? Ongoing line research has identified a range of factors that determine under which level of supervision a trainee is entrusted to perform a particular task under particular circumstances by a clinical supervisor [4, 5]. The personal qualities of individual trainees have been found to be particularly important factors [6–9]. Here, Ten Cate and Chen [2] recently used a qualitative approach to synthesize the varying entrustment-related qualities of trainees reported in the literature into five categories: agency (proactive towards work, teams, safety, and personal development), reliability (conscientious, predictable, accountable, and responsible), integrity (truthful, benevolent, and patient-centered), capability (specific knowledge, skills, experience, and situational awareness) and humility (recognizes limits, asks for help, and is receptive to feedback) (the A RICH framework). These five categories should be considered when making A RICH entrustment decision [2].

Beyond knowledge and skill aspects, many of the qualities in the A RICH framework fall into the area of trainees’ personality traits (i.e., relatively enduring patterns of interindividual differences in thinking, feeling, and behaving, e.g., some individuals are more reliable, humble, or proactive than others). Personality traits are often divided into five overarching factors (extraversion, agreeableness, conscientiousness, neuroticism, and openness). This so-called five-factor (Big Five) model of personality has demonstrated reliability and validity across numerous populations [10–12] and can be mapped to the qualities mentioned by ten Cate and Chen. That is, extraversion might be mapped to agency, consciousness to reliability, and agreeableness to integrity and humility. However, we believe that the personality traits of neuroticism and openness are not directly reflected in the A RICH framework.

Research has shown that individual differences in the Big Five traits are related to important outcomes such as learning and (academic) performance [13–16]. In medical education, the importance of personality traits for performing well in medical school and for good medical practice is increasingly recognized [17, 18]. Exploring the relationship between personality and performance in the workplace (based on the EPA framework) could complement and extend our understanding of the entrustment-related qualities of trainees as well as learning and assessment in the clinical workplace. However, the empirical relationship between specific trainee personality traits and supervisor entrustment decisions has not been examined. Such an analysis could be useful from both theoretical and practical points of view. Theoretically, this approach could show whether the aspects developed qualitatively in the A RICH framework can be supported empirically. Practically, this approach could help to identify personality traits that affect trainees’ learning and supervisors’ entrustment decisions in the workplace.

The aim of this study was to analyse the relationship between the workplace performance of final-year medical students and their personality traits, resulting in the following research question: Are individual differences in medical students’ Big Five personality traits related to their workplace performance? To address this question, personality traits and workplace performance were measured by conducting an online, cross-sectional field study. This was done using a framework consisting of levels of experienced supervision and a defined set of EPAs, both adjusted for undergraduate medical training.

## Methods

### Setting

This study was conducted as part of the German national research project “Studierendenauswahlverbund” (STAV) on the selection of medical students for entry into medical school [19]. In Germany, undergraduate medical education programs take six years to complete. The first 5 years cover basic and clinical sciences, followed by a final clerkship year with three rotations (3.5 months each): one rotation in internal medicine, one rotation in surgery and one elective rotation. The aim of the final clerkship year is for medical students to actively participate in the clinical workplace under the instruction, supervision and responsibility of a physician [20]. There are currently no mandatory formative or summative workplace assessments in place during the final clerkship year.

### Study design

This was an online-administered, cross-sectional field study. The study protocol was approved by the local psychological ethics committee (LPEK) at the Center for Psychosocial Medicine at the University Medical Center Hamburg-Eppendorf (application number: LPEK-0042).

### Questionnaire development

The questionnaire, developed by the STAV research team through an iterative and collaborative process, was sent to final-year medical students in Germany. The questionnaire covered sociodemographic characteristics, personality traits and workplace performance in professional activities, among other reported content areas. The questionnaire was piloted by members of the STAV research team.

### Personality traits

Personality traits were measured via self-reports using the German Big Five Inventory-SOEP (BFI-S) [21]. This instrument is based on the BFI [22] and was developed as a short economic version for use with the Socio-Economic Panel (SOEP). The scale consists of a total of 15 items and measures extraversion, agreeableness, conscientiousness, neuroticism, and openness, all measured with three items each. The BFI-S items are rated on a 7-point Likert scale ranging from 1 (“does not apply at all”) to 7 (“applies perfectly”).

### Workplace performance measured using an EPA framework

Workplace performance refers to the number of professional activities that a trainee can carry out and the level of supervision needed to carry out the respective professional activity. As the basis, a set of 12 core end-of-training EPAs previously developed for undergraduate medical education students at the Charité - Universitätsmedizin Berlin, Germany, was used [23]. The EPA descriptions used in the survey are shown in Table 1.

**Table 1:** Descriptions of the 12 core end-of-undergraduate medical training EPAs as elaborated in the survey

1.	Take a medical history, perform a physical examination and summarize the results in a structured manner (typical presentations and/or common disease/complaint patterns)
2.	Compile a diagnostic plan and initiate implementation (common and typical complaints, findings and clinical pictures, stepwise diagnostics)
3.	Interpret test results and initiate further steps (commonly used tests)
4.	Compile a treatment plan and initiate implementation (common diseases, typical courses)
5.	Perform general procedures of a physician (at least 5 of the following 7 medical procedures: venous blood sampling, insertion of a peripheral access [venule], taking a blood culture, taking a smear, administering an infusion, applying simple dressings, recording a 12-lead ECG)
6.	Seek consent for medical procedures and diagnostics (informing about the procedure, benefits, risks and possible alternatives)
7.	Inform and advise patients (common counselling reasons and complaint pattern)
8.	Present a patient history (structured; according to target person[s] and situation requirements)
9.	Give or receive a patient handover (structured; according to target person[s] and situation requirements)
10.	Write and transmit a patient report (structured; make or initiate report transmission)
11.	Recognize an emergency situation and act upon it (roughly estimate the criticalness, provide immediate medical assistance, call for help)
12.	Undertake an evidence-based patient case and initiate patient-specific implementation (application of evidence-based medicine, including literature search)

Participants were asked to indicate the level of supervision while carrying out each of the respective professional activity at least 3 times. The levels of supervision were adapted to undergraduate medical education [24]. The 5 levels were as follows:

Level 1: I did not perform the task.

Level 2: I carried out this task in co-activity with a supervisor.

Level 3: I carried out this task autonomously, and all findings were double-checked.

Level 4: I carried out this task autonomously, and key findings were double-checked.

Level 5: I carried out this task autonomously, and key findings were discussed afterwards.

The questionnaire was administered using the online software LimeSurvey (version 2.62.2; Limesurvey GmbH, Hamburg, Germany). Final-year medical students from all German medical faculties were invited by email via the listservs of the respective medical faculties. Invitations were sent to reach students within or shortly after their last rotation in the final clerkship year. Before data collection, the students were informed in writing about the purpose of the study and had to provide informed consent.

### Statistical analysis

The statistical analysis was performed using SPSS (version 29.0; IBM, Ehningen, Germany; Armonk, Released 2022). The SPSS analysis code can be found in Appendix 1. Descriptive statistics were calculated for sample size, participant age and gender, the scores on the BFI-S dimensions and the level of supervision for each end-of-undergraduate medical training EPA.

Inferential statistical analysis included the calculation of Cronbach’s alpha, Pearson and Spearman correlations and multiple regression. Cronbach’s alpha was calculated for the scores on the BFI-S dimensions. Pearson correlations were computed for each EPA and each Big Five dimension to analyse the relationship between the BFI-S scores and EPA framework-based workplace performance. To check for the robustness of the results and given that the EPA scale is ordinal, we additionally computed Spearman´s rank-order correlations. Furthermore, multiple regression analyses were conducted to analyse the joint relationship between the five personality traits and the level of supervision for each EPA. For all correlations and multiple regressions, we did not include individuals (per EPAs) who reported supervision level 1. This was because we could not determine whether the students were not capable of performing the EPA or whether they had not yet had the opportunity to perform the EPA.

## Results

### Study participants

A total of 1,111 students from 35 medical faculties completed the STAV survey. The data were included in the analysis if the participating students were in their last final-year clerkship rotation or had just finished the last rotation. In total, data from 880 participants were included in the study. The mean age of the included final-year medical students was 27.2 years (SD = 3.0), 568 (65%) students were female, and 312 (35%) students were male.

#### Characteristics of the personality traits

The descriptive statistics as well as the Cronbach’s coefficient for the respective Big Five dimension are shown in Table 2.

**Table 2:** The descriptive statistics and the Cronbach’s coefficient for the Big Five dimension in the survey

	Mean (SD)	Cronbach’s coefficient
Extraversion	4.95 (1.19)	0.77
Agreeableness	5.76 (0.87)	0.60
Conscientiousness	5.84 (0.83)	0.57
Neuroticism	3.78 (1.24)	0.74
Openness	4.73 (1.16)	0.61

### Characteristics of workplace performance using an EPA framework

**Fig. 1:**
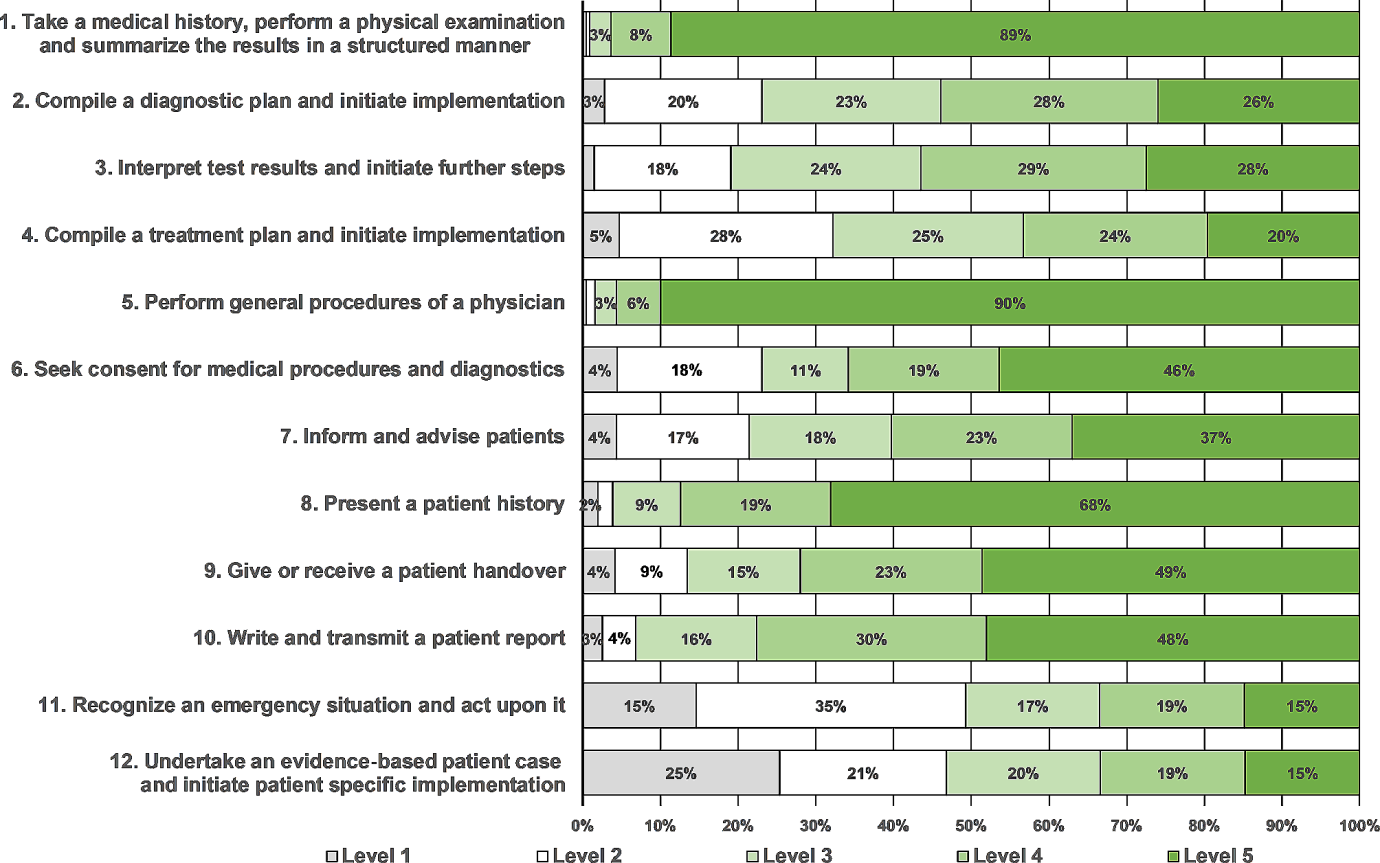
Frequency of levels of supervision (levels 1–5) under which final-year clerkship students carried out end-of-undergraduate medical training EPAs. Frequencies are expressed as a percentage of all reported levels of supervision for each EPA. The supervision levels ranged from “I did not perform the task” (level 1) to “I carried out this task autonomously, and key findings were discussed afterwards” (level 5)

Figure 1 shows the variation in the levels of supervision under which the final year clerkship students carried out the 12 EPAs. Nine of the 12 professional activities were performed by 75% of the students under supervision levels 3–5, i.e., autonomously without direct supervision. These EPAs ranged from “take a medical history, perform a physical examination and summarize the results in a structured manner” (99%) to “write and transmit a patient report” (93%). The frequencies of supervision levels without direct supervision (levels 3–5) were lower for “recognize an emergency situation and act upon it” (51%), “undertake an evidence-based patient case and initiate patient-specific implementation” (53%), and “compile a treatment plan and initiate implementation” (68%). The levels of supervision correlated significantly amongst all EPAs (see the Appendix 2).

### Relationship between the personality traits and workplace performance using an EPA framework

Significant Pearson correlations were found between the level of supervision under which final-year clerkship students carried out the EPAs and their scores on the BFI-S for all 5 Big Five traits (Table 3). There were 8 EPAs with significant correlations for extraversion, 4 for agreeableness, 8 for conscientiousness, 10 for neuroticism, and 4 for openness. All the correlations were positive, except that for neuroticism, which was negative. The correlations were all small to moderate in size (*r* = 0.17 to *r*= -0.23). Similar results were observed for the Spearman’s rank correlations (see the Appendix 3). In both analyses, significant correlations with at least one Big 5 dimension were found for all EPAs, except for EPA 5 (“perform general procedures of a physician”).

**Table 3:** Results of Pearson correlation analysis between the levels of supervision under which final-year clerkship students carried out end-of-undergraduate medical training EPAs and their scores on the dimensions of the Big Five Inventory-SOEP. *** *p* < 0.001; ** *p* < 0.01; * *p* < 0.05

	Extraversion	Agreeableness	Conscientiousness	Neuroticism	Openness	N
1. Take a medical history, perform a physical examination and summarize the results in a structured manner”	− 0.01	0.10**	0.06	− 0.10**	0.04	876
2. Compile a diagnostic plan and initiate implementation	0.11**	0.02	0.10**	− 0.21***	0.03	855
3. Interpret test results and initiate further steps	0.12***	0.02	0.12***	− 0.21***	0.05	867
4. Compile a treatment plan and initiate implementation	0.12***	0.02	0.13***	− 0.23***	0.04	839
5. Perform general procedures of a physician	− 0.02	0.06	0.04	0.02	− 0.02	876
6. Seek consent for medical procedures and diagnostics	0.08*	0.02	− 0.01	− 0.06	0.09*	841
7. Inform and advise patients	0.09**	0.02	0.01	− 0.13***	0.10**	842
8. Present a patient history	0.04	0.12***	0.13***	− 0.08*	0.03	863
9. Give or receive a patient handover	0.08*	0.08*	0.09**	− 0.12***	0.02	844
10. Write and transmit a patient report	0.05	0.08*	0.12***	− 0.16***	0.04	858
11. Recognize an emergency situation and act upon it	0.09*	0.00	0.08*	− 0.17**	0.09*	752
12. Undertake an evidence-based patient case and initiate patient-specific implementation	0.16***	− 0.01	0.17***	− 0.20***	0.10**	657

Multiple regression analysis also revealed that the BFI-S dimension scores significantly predicted workplace performance for 11 of the 12 EPAs (excluding EPA 5; see Table 4). Here, the amount of explained variance varied across the EPAs, ranging from 0.8% (EPA 6: “seek consent for medical procedures and diagnostics”) to 7.5% (EPA 12: “undertake an evidence-based patient case discussion and initiate patient-specific implementation”. Across all EPAs, the conscientiousness and neuroticism dimension scores were regularly associated with the level of supervision.

**Table 4:** Multiple regression analysis of the levels of supervision under which final-year clerkship students carried out the 12 end-of-undergraduate medical training EPAs and their scores on the 5 dimensions of the Big Five Inventory-SOEP. Legend: ΔR^2 =^ adjusted R^2^. B = unstandardised regression coefficient. SE = standard error. β = standardised regression coefficient. ***p < 0.001. **p < 0.01. *p < 0.05

EPA		Model			Regression coefficients				
		R^2^	ΔR^2^	F		B	SE	β	t
1.	Take a medical history, perform a physical examination and summarize the results in a structured manner	0.025	0.019	4.393***	Extraversion	− 0.023	0.014	− 0.060	-1.657
					Agreeableness	0.047	0.019	0.090	2.518*
					Conscientiousness	0.018	0.020	0.032	0.906
					Neuroticism	− 0.042	0.013	− 0.113	-3.238**
					Openness	0.013	0.014	0.032	0.916
2.	Compile a diagnostic plan and initiate implementation	0.053	0.048	9.580***	Extraversion	0.037	0.033	0.041	1.137
					Agreeableness	− 0.013	0.044	− 0.011	− 0.305
					Conscientiousness	0.103	0.047	0.078	2.201*
					Neuroticism	− 0.169	0.031	− 0.192	-5.504***
					Openness	0.006	0.033	0.007	0.193
3.	Interpret test results and initiate further steps	0.057	0.052	10.478***	Extraversion	0.039	0.032	0.044	1.224
					Agreeableness	− 0.024	0.043	− 0.020	− 0.568
					Conscientiousness	0.128	0.045	0.100	2.829**
					Neuroticism	− 0.161	0.030	− 0.187	-5.405***
					Openness	0.016	0.031	0.017	0.506
4.	Compile a treatment plan and initiate implementation	0.065	0.059	11.566***	Extraversion	0.036	0.034	0.039	1.072
					Agreeableness	− 0.030	0.045	− 0.024	− 0.663
					Conscientiousness	0.140	0.047	0.105	2.954*
					Neuroticism	− 0.183	0.031	− 0.206	-5.860***
					Openness	0.010	0.034	0.010	0.294
5.	Perform general procedures of a physician	0.007	0.001	1.236	Extraversion	− 0.010	0.015	− 0.023	− 0.631
					Agreeableness	0.034	0.020	0.060	1.664
					Conscientiousness	0.022	0.022	0.036	1.009
					Neuroticism	0.008	0.014	0.021	0.588
					Openness	− 0.013	0.015	− 0.030	− 0.862
6.	Seek consent for medical procedures and diagnostics	0.014	0.008	1.172*	Extraversion	0.050	0.036	0.050	1.358
					Agreeableness	0.009	0.049	0.007	0.186
					Conscientiousness	− 0.044	0.052	− 0.031	− 0.843
					Neuroticism	− 0.047	0.034	− 0.049	-1.365
					Openness	0.076	0.036	0.075	2.098*
7.	Inform and advise patients	0.026	0.020	4.468***	Extraversion	0.038	0.035	0.040	1.090
					Agreeableness	0.015	0.047	0.011	0.312
					Conscientiousness	− 0.030	0.049	− 0.022	− 0.612
					Neuroticism	− 0.102	0.033	− 0.111	-3.115**
					Openness	0.082	0.035	0.084	2.367*
8.	Present patient history	0.029	0.024	5.175***	Extraversion	0.000	0.022	0.000	− 0.011
					Agreeableness	0.078	0.030	0.094	2.613**
					Conscientiousness	0.085	0.032	0.096	2.672**
					Neuroticism	− 0.039	0.021	− 0.066	-1.878
					Openness	− 0.001	0.022	− 0.001	− 0.034
9.	Give or receive a patient handover	0.025	0.019	4.307***	Extraversion	0.029	0.031	0.035	0.940
					Agreeableness	0.070	0.042	0.060	1.665
					Conscientiousness	0.072	0.044	0.059	1.635
					Neuroticism	− 0.083	0.029	− 0.101	-2.837**
					Openness	− 0.005	0.031	− 0.005	− 0.154
10.	Write and transmit a patient report	0.041	0.035	7.247***	Extraversion	− 0.016	0.027	− 0.022	− 0.611
					Agreeableness	0.051	0.036	0.051	1.418
					Conscientiousness	0.100	0.038	0.094	2.631**
					Neuroticism	− 0.110	0.025	− 0.156	-4.442***
					Openness	0.013	0.026	0.018	0.504
11.	Recognize an emergency situation and act upon it	0.042	0.036	6.530***	Extraversion	0.024	0.037	0.025	0.654
					Agreeableness	− 0.049	0.050	− 0.037	− 0.975
					Conscientiousness	0.082	0.053	0.059	1.567
					Neuroticism	− 0.148	0.035	− 0.158	-4.223***
					Openness	0.077	0.037	0.078	2.099*
12.	Undertake an evidence-based patient case discussion and initiate patient-specific implementation	0.082	0.075	11.685***	Extraversion	0.077	0.038	0.082	2.043*
					Agreeableness	− 0.093	0.051	− 0.072	-1.810
					Conscientiousness	0.206	0.052	0.157	3.927***
					Neuroticism	− 0.145	0.035	− 0.162	-4.153***
					Openness	0.081	0.037	0.084	2.187*

## Discussion

Worldwide, medical curricula are increasingly structured around the entrustment of EPAs [25–27]. Understanding the factors that influence entrustment decisions is important, as they have the potential to stimulate transparent discussion and reflection between supervising doctors and trainees. Personal qualities of trainees play a key role, and this study complements and extends previous, mostly qualitative, research on the personality traits that are related to the decisions to entrust trainees with professional patient-care tasks. The final-year medical students varied widely in the extent to which they had actually performed a set of end-of-undergraduate medical training EPAs, and this variation showed no ceiling effect. Using a quantitative, empirical approach, we found that individual differences in the Big Five traits were related to the level of supervision under which final-year clerkship students carried out end-of-undergraduate medical training EPAs. For the personality traits, the highest number of positive correlations was found for (low) neuroticism, conscientiousness, and extraversion. That is, trainees who reported being relaxed and without worry (i.e., low neuroticism), thorough and efficient (i.e., conscientiousness), and communicative and sociable (i.e., extraversion) were generally able to carry out EPAs with less supervision and greater autonomy. The findings and implications of this study in the context of the literature is discussed in the following sections.

The results of this study showed that the favorable Big Five traits were associated with better performance in almost all EPAs, accounting for between 0.8% and 7.5% of the variance in levels of supervision as a measure of workplace performance. The strongest effects of personality traits were found for EPA 12 (“undertake an evidence-based patient case discussion and initiate patient specific implementation”), EPA 4 (“compile a treatment plan and initiate implementation”), and EPA 3 (“interpret test results and initiate further steps”). Weaker effects were found for the other EPAs where the majority of trainees reached the highest level of supervision and for which there was low variance. These findings further support the assumption that the personality traits of trainees play an important role in their workplace performance [2, 6–9].

Of all the Big Five traits, individual differences in neuroticism showed the highest number of correlations with the levels of experienced supervision for the performance of the end-of-undergraduate medical training EPAs. Individuals high in neuroticism tend to become anxious, worried and irritable more easily or intensely than others [28]. In the context of busy clinical workplaces, this personality trait may translate into difficulties coping with stress and anxiety, responding to feedback and criticism and solving problems, especially under stress, which can affect individuals’ ability to perform professional tasks effectively. It could also be that students with high neuroticism levels were less self-confident and sought more reassurance when performing tasks. Although these findings are in line with a meta-analysis highlighting the negative relationship between neuroticism and job performance [16], the relevance of neuroticism is a new finding in the area of entrustment decision-making.

The Big Five dimensions of extraversion and conscientiousness showed positive correlations with the levels of experienced supervision in a high number of the 12 EPAs, indicating higher levels of trainee autonomy. The personality trait of conscientiousness refers to individuals who tend to be highly thoughtful, organized, mindful of details, and responsible, indicating impulse control and goal-directed behaviours. The extraversion dimension refers to talkativeness, assertiveness, and energy. For conscientiousness, these findings are in line with previous research, highlighting trainee conscientiousness as a central factor in entrustment decision-making, particularly early work by Kennedy and colleagues [6]). The high number of correlations between the Big 5 score for conscientiousness and the levels of supervision across the EPAs is also consistent with previous research on personality traits and academic achievement and job performance across many occupational sectors [13]. In addition to the personality traits of extraversion, conscientiousness, and neuroticism, we found positive correlations between the levels of experienced supervision and the Big Five agreeableness and openness dimension scores, although these were lower in number and related to individual EPAs.

Our findings provide some support for the A RICH framework [2]. That is, the three dimensions of extraversion, conscientiousness, and agreeableness overlap with the qualities of integrity, reliability, humility, and agency. However, agreeableness was significant for only a few EPAs, although it is prominently represented by two qualities in the A Rich framework. Moreover, neuroticism, which was found to be the most important predictor of workplace involvement, is not (yet) covered by the A RICH framework on trainee qualities that enable entrustment decisions by clinical supervisors [2].

This study has several implications. Individual differences in personality traits were considered as a new perspective for understanding the factors that clinical supervisors weigh in their decisions to entrust trainees with responsibility in patient care. Future research could extend this research, for instance, by investigating the relationship between personality traits and the explicit level of entrustment that trainees receive in the workplace, studying other trainee cohorts and different clinical contexts, and focusing on specific facets of personality (e.g., investigating which part of an individual´s neuroticism affects entrustment decisions). Further lines of research may investigate how the personality traits of clinical supervisors influence their entrustment decisions and how the interplay between the personality traits of both trainees and supervisors affects entrustment decision-making. A particularly relevant finding is the strong role of neuroticism in entrustment decisions, which should be further explored in future quantitative and qualitative research. These findings also have implications for the training and development of medical trainees. Although there are already approaches to selecting and training (future) medical students regarding emotional stability [29, 30], the findings suggest that even more attention should be given to the emotional stability (or lack thereof) of students and physicians.

This study has some limitations. First, although we managed to recruit a large sample, the context in which the participating students were evaluated was the final clerkship year in Germany. Therefore, future research should investigate the transferability of these findings to other contexts and countries. Second, the study was not based on explicit entrustment decisions by clinical supervisors but rather on the levels of supervision experienced in the workplace and reported by the students. This may have made it unclear whether the supervisors had less confidence in the trainees and therefore preferred to “double check” or whether the trainees themselves had less confidence and therefore preferred to “double check”. Future research should disentangle these two (not mutually exclusive) explanations. Third, the BFI measure is based on a short scale, which has lower internal consistency than longer scales and did not allow us to account for facet differences within the Big Five dimensions. Here, future studies could use longer scales to assess each dimension more thoroughly.

In conclusion, this study introduced personality traits as a new perspective for understanding the role of individual trainees’ personal qualities in clinical supervisors’ decision-making. We found that the level of supervision on a set of end-of undergraduate medical training EPAs was related to the personality traits of final-year medical students. In relation to the existing body of research, the results of this study confirm the role of conscientiousness in entrustment decision-making. In addition, the findings indicate that the personality trait of neuroticism should be considered as an important trainee quality in the context of entrustment decision-making.

### Electronic supplementary material

Below is the link to the electronic supplementary material.

Supplementary Material 1

Supplementary Material 2

Supplementary Material 3

## Data Availability

The data of this study will be made available from the corresponding author upon reasonable request.
