# Supplementary material for: Big five personality traits of medical students and workplace performance in the final clerkship year using an EPA framework
Source: BMC Med Educ. 2024 Apr 25;24:453. doi: 10.1186/s12909-024-05434-x (PMC11044476; doi:10.1186/s12909-024-05434-x)
Supplement: Supplementary file 2 — Supplementary Material 2 [file 12909_2024_5434_MOESM2_ESM.pdf]

Appendix to “Big Five personality traits of medical students and workplace performance in the final clerkship year using an EPA framework” (2024). Harm Peters, Amelie Garbe, Simon M. Breil, Sebastian Oberst, Susanne Selch, and Ylva Holzhausen.

EPA	1	2	3	4	5	6	7	8	9	10	11	12
**1.** Take a medical history, perform a physical examination and summarize the results in a structured manner"	1	.23**	.24**	.18**	.42**	.20**	.23**	.41**	.27**	.33**	.13**	.17**
**2.** Compile a diagnostic plan and initiate implementation		1	.75**	.76**	.13**	.14**	.29**	.30**	.33**	.36**	.34**	.41**
**3.** Interpret test results and initiate further steps			1	.78**	.11**	.15**	.31**	.29**	.37**	.36**	.32**	.40**
**4.** Compile a treatment plan and initiate implementation				1	.10*	.15**	.33**	.29**	.36**	.35**	.32**	.42**
**5.** Perform general procedures of a physician					1	.14**	.14**	.31**	.22**	.26**	.13**	.11*
**6.** Seek consent for medical procedures and diagnostics						1	.45**	.13**	.12**	.18**	.18**	.13**
**7.** Inform and advise patients							1	.26**	29**	.28**	.31**	.32**
**8.** Present a patient history								1	.52**	.44**	.21**	.31**
**9.** Give or receive a patient handover									1	.44**	.31**	.38**
**10.** Write and transmit a patient report										1	.24**	.34**
**11.** Recognize an emergency situation and act upon it											1	.42**
**12.** Undertake an evidence-based patient case and initiate patient-specific implementation												1

Appendix 2: Results of Pearson correlations between the respective EPAs. ** p<0.001; * p<0.01
