# Supplementary material for: Big five personality traits of medical students and workplace performance in the final clerkship year using an EPA framework
Source: BMC Med Educ. 2024 Apr 25;24:453. doi: 10.1186/s12909-024-05434-x (PMC11044476; doi:10.1186/s12909-024-05434-x)
Supplement: Supplementary file 3 — Supplementary Material 3 [file 12909_2024_5434_MOESM3_ESM.pdf]

Appendix to “Big Five personality traits of medical students and workplace performance in the final clerkship year using an EPA framework” (2024). Harm Peters, Amelie Garbe, Simon M. Breil, Sebastian Oberst, Susanne Selch, and Ylva Holzhausen.

Analysis code SPSS

Recoding & Computing Big 5 Variables

```
DATASET ACTIVATE DataSet1.
RECODE BFI03 BFI06 BFI08 BFI15 (1=7) (2=6) (3=5) (4=4) (5=3) (6=2) (7=1) INTO BFI03N BFI06N BFI08N
  BFI15N.
EXECUTE.
```

```
DATASET ACTIVATE DataSet1.
COMPUTE Gewissenhaftigkeit=(Gew1+NGew8+Gew12) / 3.
EXECUTE.

COMPUTE Extraversion=(Extra2+NExtra6+Exra9) / 3.
EXECUTE.

COMPUTE Verträglichkeit=(NVertr3+Vertr7+Vertr13) / 3.
EXECUTE.

COMPUTE Offenheit=(Offen4+Offen10+Offen14) / 3.
EXECUTE.

COMPUTE Neurotizismus=(Neuro5+Neuro11+NNeuro15) / 3.
EXECUTE.
```

Reliability of Big 5 Variables

```
RELIABILITY
  /VARIABLES=Gew1 NGew8 Gew12
  /SCALE('ALL VARIABLES') ALL
  /MODEL=ALPHA.

RELIABILITY
  /VARIABLES=Extra2 NExtra6 Exra9
  /SCALE('ALL VARIABLES') ALL
  /MODEL=ALPHA.

RELIABILITY
  /VARIABLES=NVertr3 Vertr7 Vertr13
  /SCALE('ALL VARIABLES') ALL
  /MODEL=ALPHA.

RELIABILITY
  /VARIABLES=Offen4 Offen10 Offen14
  /SCALE('ALL VARIABLES') ALL
  /MODEL=ALPHA.

RELIABILITY
  /VARIABLES=Neuro5 Neuro11 NNeuro15
  /SCALE('ALL VARIABLES') ALL
  /MODEL=ALPHA.
```

Descriptive statistics

```
FREQUENCIES VARIABLES=EPA01 EPA02 EPA03 EPA04 EPA05 EPA06 EPA07 EPA08 EPA09 EPA10 EPA11 EPA12
  /ORDER=ANALYSIS.

DESCRIPTIVES VARIABLES=Gewissenhaftigkeit Extraversion Verträglichkeit Offenheit Neurotizismus
  /STATISTICS=MEAN STDDEV MIN MAX.

DESCRIPTIVES VARIABLES=Alter
  /STATISTICS=MEAN STDDEV MIN MAX.

FREQUENCIES VARIABLES=Geschlecht
  /ORDER=ANALYSIS.
```

Correlational analyses

```
CORRELATIONS
  /VARIABLES=EPA01 EPA02 EPA03 EPA04 EPA05 EPA06 EPA07 EPA08 EPA09 EPA10 EPA11 EPA12
    Gewissenhaftigkeit Neurotizismus Extraversion Verträglichkeit Offenheit
  /PRINT=TWOTAIL NOSIG FULL
  /MISSING=PAIRWISE.
NONPAR CORR
  /VARIABLES=EPA01 EPA02 EPA03 EPA04 EPA05 EPA06 EPA07 EPA08 EPA09 EPA10 EPA11 EPA12
    Gewissenhaftigkeit Neurotizismus Extraversion Verträglichkeit Offenheit
  /PRINT=SPEARMAN TWOTAIL NOSIG FULL
  /MISSING=PAIRWISE.
```

Regression analyses

```
REGRESSION
  /MISSING LISTWISE
  /STATISTICS COEFF OUTS R ANOVA
  /CRITERIA=PIN(.05) POUT(.10)
  /NOORIGIN
  /DEPENDENT EPA01
  /METHOD=ENTER Gewissenhaftigkeit Neurotizismus Extraversion Verträglichkeit Offenheit.

REGRESSION
  /MISSING LISTWISE
  /STATISTICS COEFF OUTS R ANOVA
  /CRITERIA=PIN(.05) POUT(.10)
  /NOORIGIN
  /DEPENDENT EPA02
  /METHOD=ENTER Gewissenhaftigkeit Neurotizismus Extraversion Verträglichkeit Offenheit.

REGRESSION
  /MISSING LISTWISE
  /STATISTICS COEFF OUTS R ANOVA
  /CRITERIA=PIN(.05) POUT(.10)
  /NOORIGIN
  /DEPENDENT EPA03
  /METHOD=ENTER Gewissenhaftigkeit Neurotizismus Extraversion Verträglichkeit Offenheit.
```

```
REGRESSION
 /MISSING LISTWISE
 /STATISTICS COEFF OUTS R ANOVA
 /CRITERIA=PIN(.05) POUT(.10)
 /NOORIGIN
 /DEPENDENT EPA04
 /METHOD=ENTER Gewissenhaftigkeit Neurotizismus Extraversion Verträglichkeit Offenheit.

REGRESSION
 /MISSING LISTWISE
 /STATISTICS COEFF OUTS R ANOVA
 /CRITERIA=PIN(.05) POUT(.10)
 /NOORIGIN
 /DEPENDENT EPA05
 /METHOD=ENTER Gewissenhaftigkeit Neurotizismus Extraversion Verträglichkeit Offenheit.

REGRESSION
 /MISSING LISTWISE
 /STATISTICS COEFF OUTS R ANOVA
 /CRITERIA=PIN(.05) POUT(.10)
 /NOORIGIN
 /DEPENDENT EPA06
 /METHOD=ENTER Gewissenhaftigkeit Neurotizismus Extraversion Verträglichkeit Offenheit.

REGRESSION
 /MISSING LISTWISE
 /STATISTICS COEFF OUTS R ANOVA
 /CRITERIA=PIN(.05) POUT(.10)
 /NOORIGIN
 /DEPENDENT EPA07
 /METHOD=ENTER Gewissenhaftigkeit Neurotizismus Extraversion Verträglichkeit Offenheit.

REGRESSION
 /MISSING LISTWISE
 /STATISTICS COEFF OUTS R ANOVA
 /CRITERIA=PIN(.05) POUT(.10)
 /NOORIGIN
 /DEPENDENT EPA08
 /METHOD=ENTER Gewissenhaftigkeit Neurotizismus Extraversion Verträglichkeit Offenheit.

REGRESSION
 /MISSING LISTWISE
 /STATISTICS COEFF OUTS R ANOVA
 /CRITERIA=PIN(.05) POUT(.10)
 /NOORIGIN
 /DEPENDENT EPA09
 /METHOD=ENTER Gewissenhaftigkeit Neurotizismus Extraversion Verträglichkeit Offenheit.

REGRESSION
 /MISSING LISTWISE
 /STATISTICS COEFF OUTS R ANOVA
 /CRITERIA=PIN(.05) POUT(.10)
 /NOORIGIN
 /DEPENDENT EPA10
 /METHOD=ENTER Gewissenhaftigkeit Neurotizismus Extraversion Verträglichkeit Offenheit.
```

```
REGRESSION
  /MISSING LISTWISE
  /STATISTICS COEFF OUTS R ANOVA
  /CRITERIA=PIN(.05) POUT(.10)
  /NOORIGIN
  /DEPENDENT EPA11
  /METHOD=ENTER Gewissenhaftigkeit Neurotizismus Extraversion Verträglichkeit Offenheit.
```

```
REGRESSION
  /MISSING LISTWISE
  /STATISTICS COEFF OUTS R ANOVA
  /CRITERIA=PIN(.05) POUT(.10)
  /NOORIGIN
  /DEPENDENT EPA12
  /METHOD=ENTER Gewissenhaftigkeit Neurotizismus Extraversion Verträglichkeit Offenheit.
```
